# The King to His People

**Published:** 1911-11-11

**Authors:** 


### THE KING TO HIS PEOPLE.

Under this title Messrs. Williams and Norgate publish this week an interesting volume which contains the most important speeches delivered by King George both as Prince of Wales and since his accession to the throne.* To those of us who are connected with charitable institutions, and to those others who are more actively interested in the profession, the study, and the teaching of medicine, it is indeed no news to hear that King George has always shown himself in cordial sympathy, often expressed in ways that are very practical and stimulating, with the routine duties in which we are engaged. But it is possible that only comparatively few realise how knowledgeable that interest has been and is. It is difficult for one man to grasp and to hold the increasingly rapid development of modern institutional and medical work, and it is not to be expected that King George can deal in an expert fashion with the manifold problems which confront the institutional and medical worker. But he has shown that he can fully appreciate and deal with the problems which are of a pressing and immediate character, and that he has the ability, the energy, and, be it added, the sympathy to deal with them in a fashion that may serve as a model for those of us who find our time so limited that we centre our activities round a comparatively small nucleolus of interest.

To institutional and medical workers this compilation of the King's speeches, therefore, furnishes much that is instructive, and a great deal more that is encouraging, helpful, and a testimony to the fact that in our endeavours to alleviate what Keats has called "the giant agony of the world" we have with us in our struggle the active sympathy of our Sovereign, and that this sympathy is not founded merely on the sentiment that underlies the average man's interest in hospitals, but on a thorough understanding of the difficulties we have to face and the character of the work that we have to do. Abundant evidence of this expert knowledge is furnished in the speeches. Addressing the Fellows of the Royal Society on the occasion of his admission to the Fellowship, the Prince of Wales (as he then was) remarked that he could assure the Society of his hearty sympathy "with that scientific study and research which now, more than ever, has become so important an essential in our national life." Opening the National Physical Laboratory in 1902, he delivered a speech which is one of the most informative in the compilation, and if we want further evidence of the attention that King George has bestowed on medical and scientific questions of the day, we find it amply recorded in the addresses given at the opening of the International Statistical Institute in 1905; the centenary dinner of the old Medical Chi in the same year; at the Battersea Polytechnic in 1904; at the Royal Medical Benevolent College, Epsom; at the dinner in aid of the National Association for Sanatoria in 1907; at the Committee meeting of the Imperial Cancer Research Fund; and on other occasions, all of which are recorded in this book. His interest in, and sympathy for institutional workers found eloquent expression in two speeches which deserve the earnest attention of hospital workers in this country. The first was spoken in 1902 when he opened the Raphael Nurses' Home at Guy's Hospital; the second five years later when he opened the new out-patients' department of St. Bartholomew's Hospital. Short as the latter is, it shows that his Royal Highness had fully grasped the essentials of modern hospital requirements. It is encouraging to hospital workers to read these extracts from his speeches; the chord that he touches is one generally of hopeful optimism, for his Majesty well realises the invaluable asset which the nation holds in its possession of the voluntary hospitals, and the fact that the greater part of the charitable public supports, and will continue to support, the system which, under able management, has done so much towards the relief of sickness and distress.

* *The King to His People*: Being the Speches and Messages of His Majesty King George V. as Prince and Sovereign. Published by permission. London: Williams and Norgate. Price 5s. net.

